# Bioconjugation of a PNA Probe to Zinc Oxide Nanowires for Label-Free Sensing

**DOI:** 10.3390/nano11020523

**Published:** 2021-02-18

**Authors:** Teresa Crisci, Andrea Patrizia Falanga, Maurizio Casalino, Nicola Borbone, Monica Terracciano, Giovanna Chianese, Mariano Gioffrè, Stefano D’Errico, Maria Marzano, Ilaria Rea, Luca De Stefano, Giorgia Oliviero

**Affiliations:** 1 Institute of Applied Sciences and Intelligent Systems, Unit of Naples, National Research Council, 80131 Naples, Italy; teresa.crisci@na.isasi.cnr.it (T.C.); maurizio.casalino@na.isasi.cnr.it (M.C.); giovanna.chianese@na.isasi.cnr.it (G.C.); mariano.gioffre@na.isasi.cnr.it (M.G.); luca.destefano@na.isasi.cnr.it (L.D.S.); 2 Department of Mathematics and Physics, University of Campania Luigi Vanvitelli, 81100 Caserta, Italy; 3 Department of Molecular Medicine and Medical Biotechnology, University of Naples Federico II, 80131 Naples, Italy; falanga@gmail.com (A.P.F.); golivier@unina.it (G.O.); 4 Department of Pharmacy, University of Naples Federico II, 80131 Naples, Italy; borbone@unina.it (N.B.); monica.terracciano@unina.it (M.T.); stefano.derrico@unina.it (S.D.); 5 Institute of Crystallography, National Research Council, 70126 Bari, Italy; maria.marzano@unina.it

**Keywords:** ZnO nanowire, PNA probe, surface functionalization, DNA, label-free biosensing

## Abstract

Zinc oxide nanowires (ZnONWs) are largely used in biosensing applications due to their large specific surface area, photoluminescence emission and electron mobility. In this work, the surfaces of ZnONWs are modified by covalent bioconjugation of a peptidic nucleic acid (PNA) probe whose sequence is properly chosen to recognize a complementary DNA (cDNA) strand corresponding to a tract of the CD5 mRNA, the main prognostic marker of chronic lymphatic leukemia. The interaction between PNA and cDNA is preliminarily investigated in solution by circular dichroism, CD melting, and polyacrylamide gel electrophoresis. After the immobilization of the PNA probe on the ZnONW surface, we demonstrate the ability of the PNA-functionalized ZnONW platform to detect cDNA in the μM range of concentration by electrical, label-free measurements. The specificity of the sensor is also verified against a non-complementary DNA sequence. These preliminary results highlight the potential application of PNA-bioconjugated ZnONWs to label-free biosensing of tumor markers.

## 1. Introduction

Biosensors could be the most effective approach to solve some of the main issues concerning the conventional diagnosis providing rapid responses, requiring a lower amount of biological materials, and reducing the waiting time for results, thus allowing timely patient treatments [1,2]. In particular, DNA-based biosensors have the potentialities to fulfill all the above-mentioned requirements due to the high degree of specificity to the target molecules and the availability of several techniques enabling the transduction of the binding event into a detectable and measurable signal. However, the DNA’s lack of resistance to enzymatic degradation in biological fluids prevents its use in in vivo measurements. Over the years, it has been inferred that the development of a biosensor based on conventional DNA is limited by different probe drawbacks, such as the reduced stability under environmental conditions such as pH and temperature, and a minimum limit of 20 bases in length to reach the specificity of a binding event, making the process costly and time-consuming [3,4].

The application of advanced nucleic acids or their analogs (i.e., aptamers, peptide nucleic acids (PNAs)) for target detection in biosensor development [5,6,7] represents an alternative solution to overcome these limitations. PNAs are DNA mimicking compounds in which the negatively charged sugar-phosphate backbone is replaced by a neutral 2-aminoethyl-glycine backbone [8]. These compounds exhibit greater stability towards chemical and enzymatic degradation and generate highly stable complexes with DNA and RNA target molecules obeying the Watson–Crick binding rule. These characteristics, together with low toxicity and immunogenicity, make PNAs one of the most valid tools for biomedical applications, both in diagnostics and therapy [9,10,11,12,13,14]. Herein, the realization of a label-free PNA-based device as a new potential diagnostic tool for the rapid monitoring of chronic lymphatic leukemia (CLL) was described. 

CLL is the most common form of leukemia in western countries. Its diagnosis is established by blood counts, differential counts, a blood smear, and immunophenotyping. Additional tests may be required in case of altered results, including a bone marrow biopsy—an invasive examination with long waiting times. In this scenario, the development of a rapid and less invasive PNA-based biosensor could be very useful.

A PNA probe fully complementary to a 12-mer DNA corresponding to a tract of the CD5 mRNA—the prognostic marker for CLL—was synthesized. The CD5 mRNA to be probed was chosen by GenBank, a bioinformatics tool, thus selecting a tract of the mRNA less prone to fold into ordered secondary structures, as well as reducing the mRNA accessibility to the PNA probe. 

Bioprobe immobilization onto the transducer is probably one of the most crucial steps in biosensor manufacturing. Indeed, an effective and reproducible immobilization strictly accounts for the reliability and the consistency of the generated analytical data. It is fundamental to preserve the functional properties of the immobilized probe while ensuring its stable binding on the sensor surface throughout the analytical procedures [15]. In this paper, the biosensor was obtained by grafting the PNA through a covalent bond on the surface of a properly functionalized zinc oxide nanowire (ZnONW)-based electrical sensor, using silane-based chemistry. To our knowledge, this is the first study demonstrating the bioconjugation of a PNA to ZnONWs. The ZnONWs are a nanostructured form of ZnO, an n-type semiconductor characterized by a direct band-gap of 3.37 eV and an exciton binding energy of 60 meV [16]. The main application field of ZnONWs is biosensing due to their highly specific surface area, biocompatibility, electron mobility, and photoluminescence emission in the visible range [17,18]. An additional property of ZnONWs is that they can be synthesized on several substrates by a mild hydrothermal process requiring not very high temperatures (~90 °C), low-cost reagents, and simple equipment. The hydrothermal synthesis of ZnONWs on silicon, optical fibers, plastic, and cellulose has been demonstrated [19,20]. ZnONWs are characterized by an isoelectric point (IEP) of 9.5, much higher than the IEP of many biomolecules that can be efficiently immobilized on the positively charged surface of ZnONWs by electrostatic interaction. The presence of structural inhomogeneities, such as zinc interstitials and oxygen vacancies, allows the interaction between the surface of ZnONWs and thiol, carboxyl, and phosphoric acid groups [21]; the silanization of ZnONWs by (3-aminopropyl)triethoxysilane (APTES) was also demonstrated [22,23].

The ability of the synthesized PNA to selectively recognize the complementary DNA target (with and without chromophore) was preliminarily investigated in solution by using spectroscopic and electrophoretic techniques—circular dichroism (CD), CD melting, and polyacrylamide gel electrophoresis (PAGE). Then, the PNA was immobilized on the surface of the ZnONW sensors, and its specificity towards the DNA target sequence, rather than a non-complementary one, was assessed by label-free techniques—photoluminescence spectroscopy and electrical measurements—and fluorescence analyses using the labeled molecules. 

## 2. Experimental Section

### 2.1. Chemicals and Reagents

Unless otherwise reported, all chemicals and reagents were of analytical grade, purchased from Merck Life Science (Milano, Italy), and used without any further purification. Ultra-pure water (18 Ω·cm) purified from a Milli-Q purification system (Millipore, Bedford, MA, USA) was used to prepare all the aqueous solutions. 

### 2.2. PNA Synthesis and Analysis

The sequences of **PNA** and labeled PNA (PNA*) were summarized in Table 1. Their syntheses were performed using the 9-fluorenylmethoxycarbonyl (Fmoc) solid-phase strategy. After swelling 50 mg of 4-methylbenzhydrylamine (MBHA) resin (0.5 mmol/g) in dichloromethane (CH_2_Cl_2_) for 30 min and three washings in dimethylformamide (DMF), the resin was treated with 20% piperidine solution in DMF for 10 min. After washings in DMF (×5), two lysine couplings were performed using the following conditions: 5 equiv. Fmoc-L-Lys(MMT)-OH (MMT = monomethoxytrityl) monomer dissolved in N-methyl-2-pyrrolidone (NMP) 0.2 M, 5 equiv. 3-oxid hexafluorophosphate (HATU) dissolved in DMF 0.2 M; 5 equiv. N,N-diisopropylethylamine (DIPEA)/7.5 equiv. lutidine for 40 min at room temperature (RT). The presence of the free NH_2_ groups on the side chains of the two lysines allowed the functionalization of the ZnONW surface.

For PNA monomers reaction, 5 equiv. monomers were dissolved in N-Methyl-2-pyrrolidone (NMP) 0.2 M, 5 equiv. HATU dissolved in DMF 0.2 M and 5 equiv. DIPEA/7.5 equiv. lutidine, 40 min at RT. After each coupling step, 5% acetic anhydride (Ac_2_O) in DMF solution was used for the capping step for 10 min at RT. Subsequently, the Fmoc groups were removed by piperidine solution treatment. After the removal of the Fmoc groups, the free NH_2_ terminal groups on the last monomer of the sequence were acetylated. For the synthesis of the fluorescein isothiocyanate (FITC) labeled PNA (PNA*), two coupling cycles with the 2-[2-(Fmoc-amino)ethoxy]ethoxyacetic acid linker (Fmoc-AEEA-OH) at the terminal amino group of PNA were performed using the following conditions: 8 equiv. Fmoc-AEEA-OH dissolved in NMP (0.4 M), 8 equiv. HATU dissolved in DMF (0.4 M), and 8 equiv. DIPEA/12 equiv. lutidine. After the removal of the Fmoc groups, 5 equiv. FITC 0.2 M were solubilized in DMF/DIPEA (2.5:97.5 *v*/*v*), and the solution was put into the resin, which was gently stirred in the dark for 15 h. At the end of both syntheses, **PNA** and PNA* were cleaved from the solid support by treatment with trifluoroacetic acid (TFA)/anisole/ethanedithiol (9:1:1; *v*/*v*/*v*) for 4 h, and the raw products were precipitated with cold diethyl ether. The products were centrifugated, washed two times with diethyl ether, and lyophilized. The PNAs were obtained with a 48 to 50% overall yield for **PNA** and 40 to 45% for PNA* (95% average yield for each coupling estimated by Fmoc spectrophotometric measurements). The raw products were purified by semipreparative HPLC analyses, and purification was carried out on a Jasco HPLC system equipped with the PU-2089 Plus pump and the UV-2075 Plus UV detector (Jasco Europe, Cremella, Italy) using a 10 × 250 mm C-18 reverse-phase column (particle size 5 µm) from Merck Millipore (Billerica, MA, USA) using a linear gradient of CH_3_CN containing 0.1% (*v*/*v*) trifluoroacetic acid (TFA) in H_2_O containing 0.1% (*v*/*v*) TFA (from 0 to 100% in 45 min, flow 1.2 mL/min). After lyophilization, the amount of **PNA** and PNA* dissolved in pure water was estimated by quantitative UV with a Jasco V-530 spectrophotometer (λ = 220–310 nm, 400 nm/min scanning speed, 2.0 nm bandwidth) using the molar extinction coefficients ε = 104.4 cm^−1^ mM^−1^.

The final pure products were characterized by electrospray mass spectrometry (ESI-MS) using a 4000 QTRAP mass spectrometer (ThermoFisher Scientific, Waltham, MA, USA). **PNA**: ESI-MS (m/z) calcd. for [M + H]^+^ 3452.46, calcd. for [M + 2H]^2+^ 1726.73, found 1726.7, calcd. for [M + 3H]^3+^ 1151.49, found 1151.5; calcd. for [M + 4H]^4+^ 863.86, found 863.8 (Figure S1); PNA*: ESI-MS (m/z) calcd. for [M + H]^+^ 4089.64; calcd. for [M + 2H]^2+^ 2045.28, found 2045.2; calcd. for [M + 3H]^3+^ 1363.86, found 1363.7; calcd. for [M + 4H]^4+^ 1023.14, found 1023.1 (Figure S1).

### 2.3. DNA Synthesis and Analysis

The sequences of complementary DNA (**cDNA**), labeled complementary DNA (cDNA*), non-complementary DNA (**ctrlDNA**), and labeled non-complementary DNA (ctrlDNA*) used in this study are reported in Table 1. Oligonucleotide (ON) sequences were synthesized by solid-phase β-cyanoethyl phosphoramidite chemistry using an Expedite 8909 automated DNA synthesizer (Perseptive Biosystems, Framingham, MA, USA). CPG Universal Support (Glen Research, Sterling, VA, USA; 35 mg, 1.4 µmol) was used for DNA syntheses using the 1 µmol scale standard protocol, with the DMT-OFF option. For the cDNA* and ctrlDNA* syntheses, 6-[(3′,6′-dipivaloylfluoresceinyl)-carboxamido]-hexyl-1-O-[(2-cyanoethyl)-(N,N-diisopropyl)]-phosphoramidite (FAM; Glen Research) was introduced at the end of the ONs synthesis. Finally, the oligomers were removed from the resin and deprotected by treatment with concentrated aqueous ammonia at 55 °C for 12 h. Products were filtrated and concentrated under reduced pressure, redissolved in H_2_O, analyzed and purified by linear gradient HPLC (from 0% to 100% B in 30 min at 1.2 mL/min) on a Nucleogen SAX 1000-8/46 column (Macherey-Nagel, Düren, Germany) and then eluted with buffer A, 20 mM monosodium phosphate (NaH_2_PO_4_) aqueous solution, pH 7.0, containing 20% (*v*/*v*) acetonitrile (CH_3_CN); and buffer B, 1 M NaCl, 20 mM NaH_2_PO_4_ aqueous solution, pH 7.0, containing 20% (*v*/*v*) CH_3_CN. After purification, the ONs were desalted by C18 Sep-Pak cartridges (Waters Italia, Sesto San Giovanni, Italy) and eluted with a stepped gradient of 30, 50, and 100% CH_3_CN in H_2_O. ON concentration was assessed spectrophotometrically at λ = 260 nm and 90 °C, using the molar extinction coefficients ε = 131.6 cm^−1^ mM^−1^ for **cDNA** and cDNA*, and 95.3 cm^−1^ mM^−1^ for **ctrlDNA** and ctrlDNA*, as determined using the Sigma-Aldrich OligoEvaluator™ web tool.

### 2.4. Circular Dichroism (CD)

All DNA, PNA, and PNA/DNA (1:1) samples were prepared at 1 mM concentration in 100 mM phosphate-buffered saline (PBS), pH = 7.0. For ONs preparation, 12.5 nmol of **cDNA**, cDNA*, **ctrlDNA,** and ctrlDNA* were lyophilized and dissolved in 12.5 μL of 100 mM PBS. Heteroduplexes were obtained by mixing 12.5 nmol of lyophilized ON with 12.5 nmol of **PNA** in 12.5 μL of 100 mM PBS. All samples at 1 mM concentration were heated at 90 °C for 10 min and equilibrated at 4 °C for 24 h [12]. The resulting solutions were finally diluted to 0.3 mL with the PBS for CD analysis. A Jasco 1500 spectropolarimeter equipped with a Jasco PTC-348-WI temperature controller (Jasco Europe, Cremella, Italy) was used to acquire CD spectra in the 200 to 320 nm range at 25 °C, using 0.1 cm path-length cuvette in 100 mM PBS at the concentration of 41.6 µM. Five scans were recorded for each CD spectrum at 200 nm min^−1^ scan rate, 4 s response time, and 2 nm bandwidth. The PBS buffer baseline was subtracted, and the spectra were normalized to have zero ellipticity at 360 nm. Thermal denaturation experiments were also recorded in the temperature range of 5 to 90 °C by monitoring the CD values at 266 nm for both heteroduplexes at a heating reat of 0.5 °C/min.

### 2.5. Non-Denaturing Polyacrylamide Gel Electrophoresis (PAGE)

Non-denaturing gel electrophoreses were performed using 18% polyacrylamide gels. 1 × Tris-Borate-EDTA (TBE) buffer supplemented with 30 mM KCl, pH 7.0, was used for gel runs for 1 h, at 4 °C. All samples were loaded at 1 mM ON concentration. For gel loading, 2 µL of each sample were mixed to 8 µL of loading buffer (glycerol/1 × TBE + 30 mM KCl 1:9). Electrophoreses were carried out at a constant voltage of 120 V at 5 °C. The gels were visualized using a UV-Vis lamp in the visible range.

### 2.6. Fabrication Process of Zinc Oxide Nanowires Electrical Sensor

First, low-doped p-type Silicon (Si) substrates were ultrasonically cleaned in isopropanol and acetone for 10 min each, rinsed with deionized water (DI), and dried with a nitrogen flux. Then, dry thermal oxidation (4 h at 1100 °C) was carried out in order to grow a dielectric silicon dioxide (SiO_2_) layer on top of the Si substrate.

A ZnO seed layer was uniformly deposited on top of the SiO_2_/Si structure using a radio frequency (RF) magnetron sputtering system from a 99.999% pure ceramic ZnO target. The substrate was placed on the holder immediately before pumping the deposition chamber down to a pressure of 3 × 10^−6^ mbar. After that, an Argon (Ar) flux of 40 sccm was introduced in the chamber, and a 150 W RF power was applied to generate the required plasma. A 150 nm-thick ZnO thin film was deposited at room temperature, at a process pressure of 2.5 × 10^−2^ mbar, for 30 min of deposition time. Subsequently, ZnO nanowires (NWs) were grown by hydrothermal synthesis in an equimolar (0.1 M) solution prepared by dissolving a Zn^2+^ salt (Zn(NO_3_)_2_) working as a precursor of alkaline reagent hexamethylenetetramine (C_6_H_12_N_4_) in 200 mL DI water. This solution was first heated at 90 °C on a hot plate, provided with both a PID controller and a thermal sensor, and then the Si/SiO_2_/ZnO substrate was immersed with the top side facing down for 2 + 2 h. The hydrothermal growth process was performed in two steps, each with a fresh solution, to obtain longer NWs, and subsequently, the ZnO NWs were rinsed with DI water and dried with a nitrogen flux. 

**Figure 1:**
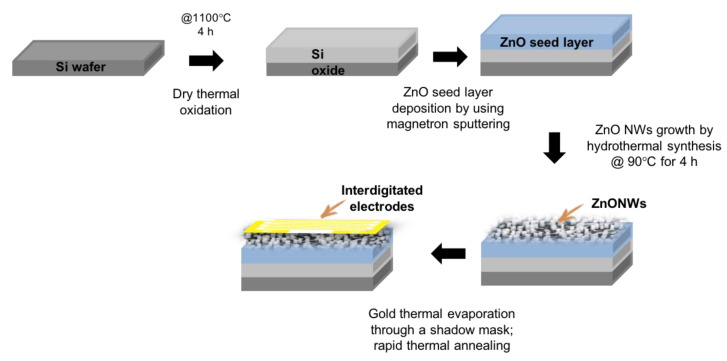
Fabrication process of the ZnONWs electrical sensor.

The interdigitated electrodes were deposited, through an iron shadow mask, on the ZnO NWs by thermal evaporation of 10 nm/300 nm-thick chromium/gold (Cr/Au) bilayer at a pressure of 4 × 10^−6^ mbar, with Cr acting as an adhesion layer. Finally, the whole structure was subjected to rapid thermal annealing at 400 °C for 5 min in a pure nitrogen atmosphere. The fabrication process was schematized in Figure 1.

### 2.7. Functionalization of ZnONWs Surface

The ZnONWs were properly functionalized to allow the covalent immobilization on their surface of the PNAs by using the free side chain NH_2_ group of lysines tail. To this aim, the functionalization procedure schematized in Figure S2 was used. (I) Hydroxyl (OH) groups were activated on the surface of ZnO NWs by exposure to oxygen plasma at 50 sccm for 40 s. (II) Samples were then silanized using a 5% APTES solution in anhydrous toluene for 30 min at room temperature. Unreacted silane was removed by intensive washings in dry toluene, and the surface was treated by a curing process performed at 110 °C for 10 min. (III) Samples were treated with a 1.7 mM bissulfosuccinimidyl suberate (BS^3^) solution in PBS, pH 7.4 at 4 °C for 4 h. BS^3^ contains an amine-reactive N-hydroxysulfosuccinimide (NHS) ester at each end of an eight-carbon spacer arm. NHS esters react with primary amines of APTES at pH 7.4 to form stable amide bonds, releasing the N-hydroxysulfosuccinimide leaving group. (IV) After washing in PBS, samples were dried by a nitrogen stream and incubated with the PNA solution in water overnight at 4 °C. (V) Unbound PNA was removed by washing the samples in PBS, and unreacted BS^3^ was passivated, incubating the devices in 1 M Tris-HCl for 20 min. 

In order to determine the PNA amount useful to saturate the surface of the ZnONWs, the FITC-labeled PNA, PNA*, was immobilized at different concentrations (25, 50, 100, and 200 μM); the saturation level of the surface was monitored by fluorescence microscopy. One-hundred μM PNA* was chosen for the hybridization experiment with the target DNA.

### 2.8. Scanning Electron Microscopy

The morphology of ZnONWs was investigated by scanning electron microscopy (SEM). SEM images were collected at 20 kV accelerating voltage by using a Carl Zeiss NTS 1500 Field Emission Scanning Electron Microscope (Carl Zeiss, Oberkochen, Germany). A secondary emission detector was used for imaging. 

### 2.9. Hybridization Experiments

The hybridization between the **PNA** probe immobilized on the ZnONWs surface and its complementary DNA sequence (**cDNA**) was obtained by dropping on the sensor surface 50 µL of increasing concentrations of **cDNA**, from 10 µM to 125 µM, in PBS 1× for 2 h, at 4 °C. After each incubation, the sample was washed by PBS + 0.2% Tween-20, PBS 1×, and water to avoid non-specific interactions. 

To demonstrate the specificity of the PNA/DNA interaction, two **PNA**-modified ZnONW sensors were incubated with the target DNA (**cDNA**) or control DNA (**ctrlDNA**) by dropping the corresponding 100 µM solution for 2 h at 4 °C. After the incubation, the samples were washed by PBS + 0.2% Tween-20, PBS 1×, and water.

The experiments were performed using both labeled and label-free DNA sequences described in Table 1.

### 2.10. Photoluminescence Spectroscopy

Steady-state photoluminescence (PL) spectra of samples were excited by a continuous wave He-Cd laser at 325 nm (KIMMON KOHA, Tokyo, Japan). The light emitted from the sample under analysis was dispersed in a SpectraPro 300i spectrometer (Princeton Instruments, Trenton, NJ, USA) and detected using a Peltier-cooled PIXIS 100F charge-coupled device (CCD) camera (Princeton Instruments, Trenton, NJ, USA). The laser line at the monochromator inlet was removed using a long-pass filter with a cut-on wavelength of 350 nm.

### 2.11. Fluorescence Microscopy

The Leica AF6000LX-DM6M-Z microscope (Leica Microsystems, Mannheim, Germany), controlled via LAS X (Leica Application Suite; rel. 3.0.13) software with a 10× objective in dry medium, was used for the fluorescence analysis of the hybridization of the FAM-labeled DNA target (DNA*) to the PNA probe. The imaging was performed using an I3 filter cube constituted by a 450 to 490 nm band-pass excitation filter, a 510 nm dichromatic mirror, and a 515 nm suppression filter.

### 2.12. Electrical Measurements

The I–V characteristics were acquired using an Agilent B2902A Source Meter (Keysight Technologies, Santa Rosa, CA, USA) driven by a PC using custom codes written in Matlab (MathWorks, Natick, MA, USA; rel. r2019a). Samples were carefully accommodated on the holder of a probe station and electrodes connected by two XYZ micromanipulators (450/360MT-6 and 550/360MT-6 series, The Micromanipulator Co., Carson City, NV, USA) provided with micrometric tips. Accurate electrical measurements were performed, at room temperature and under dark conditions, in order to show the change in resistance due to both the functionalization steps and the interaction with various concentrations of DNA. Each I–V curve is the weighted average of five curves obtained by connecting the tips in five different points of the electrodes; each of these curves is the average value of five measurements performed by varying the voltages repetitively from −5 to 5 V.

## 3. Results and Discussion

### 3.1. PNA/DNA Interaction in Solution Studies

**Figure 2:**
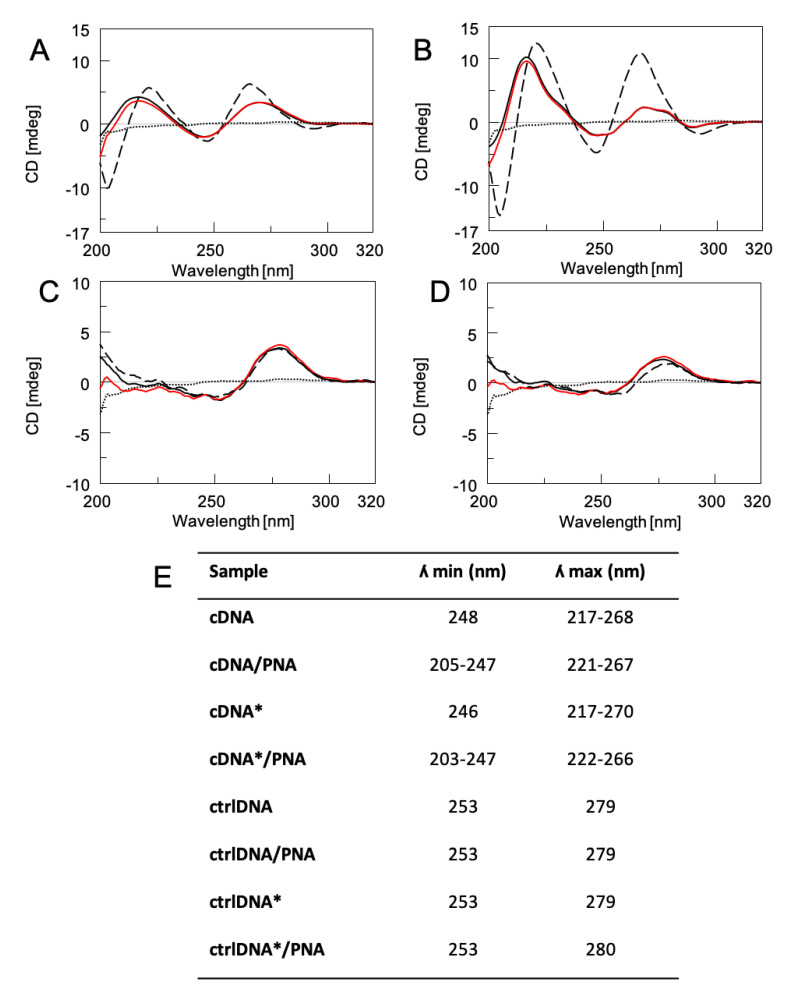
CD spectra of cDNA* and **cDNA** alone (black solid line, panel **A** and **B**, respectively) and after annealing with **PNA** (dashed line, A and B respectively); ctrlDNA* and **ctrlDNA** alone (solid black line, panel **C** and **D** respectively) and annealed with **PNA** at pH 7.0 (dashed line, **C** and **D** respectively); all samples were dissolved in 100 mM PBS. CD profiles of the arithmetic sum of each ON with **PNA** are reported as red lines. The CD spectrum of **PNA** alone is reported as a dotted line. All samples were normalized at 320 nm; Table (**E**) λ values of CD minima and maxima of each sample.

The sequence of the PNA probe is complementary to the DNA stretch corresponding to the mRNA exon codifying the CD5 glycoprotein overexpressed in the CLL cells. Solution-phase hybridization studies were performed to assess the interaction between the PNA probe and the DNA target, as well as the stability of the resulting heteroduplexes by using CD, CD melting, and PAGE techniques. The CD spectra of **PNA/cDNA**, **PNA**/cDNA*, **PNA/ctrlDNA**, and PNA/ctrlDNA* (1:1 ratio) were compared with CD profiles of **PNA**, **cDNA**, cDNA*, **ctrlDNA,** and ctrlDNA* (Figure 2), thus evaluating the formation of complexes. The CD spectra of **PNA/cDNA** and PNA/cDNA* mixtures were clearly different from that of the arithmetic sum of the individual components, thus confirming the hybridization between the **cDNA** (with and without FAM fluorophore) and the **PNA** (Figure 2A,B, respectively). Furthermore, the spectra of the two PNA/DNA mixtures showed the typical CD profile of antiparallel heteroduplexes, characterized by two positive CD bands around 260 and 220 nm and two negative CD bands around 240 and 200 nm, thus confirming the formation of the target heteroduplexes (**PNA/cDNA** and PNA/cDNA*, dashed lines panel A and B, respectively) [24,25]. Moreover, the significant reduction observed in the intensity of the positive dichroic signals at 222 and 266 nm for **PNA**/cDNA* compared to **PNA/cDNA** (Figure 2) was ascribed to the presence of the FAM fluorophore. The interaction of **PNA** with a control sequence (**ctrlDNA**), either labeled or not, was also investigated. The CCTTTTTTTTTT DNA sequence (**ctrlDNA**) was chosen with the aim of ruling out the occurrence of an aspecific binding. The **ctrlDNA** was not intended to work as a scramble DNA sequence. The spectra of **PNA**/ctrlDNA* and **PNA/ctrlDNA** (Figure 2C,D, respectively) overlapped almost perfectly with that of the arithmetic sum of the individual components, thus indicating that no hybridization occurred with the non-complementary DNA sequence, both in the presence or absence of the FAM fluorophore.

The CD melting curves of **PNA**/cDNA* and **PNA/cDNA** heteroduplexes revealed greater stability of the latter (Tm values of 40 °C and 45 °C, respectively), as also disclosed by a weak reduction in terms of the intensity of the CD signal of the FAM-labeled DNA in PNA/cDNA* heteroduplex (Figure S3). These results clearly indicated that the slight de-stabilization of the **PNA**/cDNA* heteroduplex could be ascribed to the presence of the FAM chromophore. 

The molecular size of the complexes was examined by PAGE analysis (Figure S4). In the case of the cDNA* and ctrlDNA* alone dissolved in 100 mM PBS buffer, one main band corresponding to the single strand was observed (Figure S4, lane 1 panel A and B, respectively). The PAGE mobility of the sample obtained annealing cDNA* and **PNA** (1:1) in 100 mM PBS is shown in lane 2, panel A. The single strand cDNA* band disappeared, and a new slower and clear band due to the **PNA**/cDNA* heteroduplex was observed. By contrast, no variation in the PAGE mobility of ctrlDNA* before and after its incubation with **PNA** was observed (Figure S4B, lane 1 and 2, respectively), confirming the absence of any interaction between **PNA** and the ctrlDNA* sequence.

The obtained results suggested the formation of PNA/DNA heteroduplexes only when the PNA probe was incubated in the presence of the fully complementary DNA strand. The results also evidenced a slight thermal destabilization of the heteroduplex when the **cDNA** was linked to the FAM fluorophore. However, this latter drawback would not affect the hybridization performances of the proposed PNA-functionalized ZnONWs nanodevice system, which works at room temperature and in the absence of the FAM chromophore.

### 3.2. The ZnONW Electrical Sensor: Geometry and Morphology

**Figure 3:**
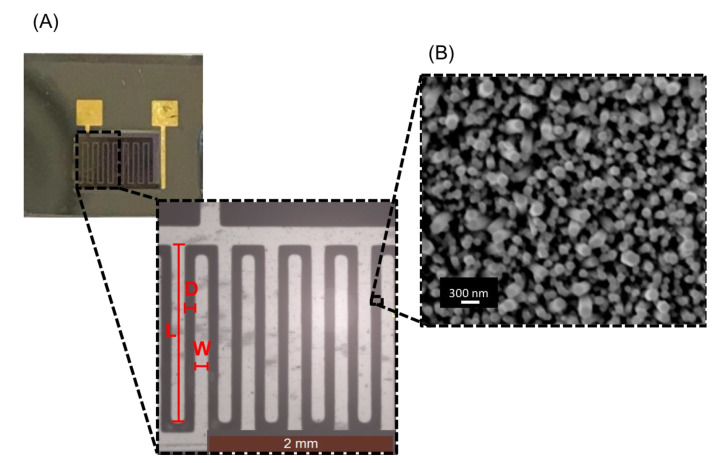
(**A**) Representative images of ZnONW-based electrical sensor. (**B**) SEM image of ZnONWs grown by hydrothermal synthesis.

In order to minimize the series resistance, a ZnONW electrical sensor was obtained, realizing interdigitated electrodes on the surface of the ZnONWs. The overall dimensions of the device were 1 × 1 cm; interdigitated electrodes were characterized by a length (L) of 2 mm, a width (W) of 0.15 mm, and a gap between electrodes (D) of 0.1 mm. Representative optical images of the sensor were reported in Figure 3A. SEM imaging performed in the region D showed a uniform layer of high-density ZnONWs, oriented in the direction perpendicular to the substrate. The width of the ZnONWs and the size of the empty spaces between the ZnONWs were measured to be 180 ± 20 nm (N = 30) and 260 ± 60 nm (N = 30), respectively (Figure 3B). The wide distributions of width and empty spaces are typical of ZnONWs obtained by hydrothermal synthesis [26].

### 3.3. Bioconjugation of PNA to the ZnONW Surface 

**Figure 4:**
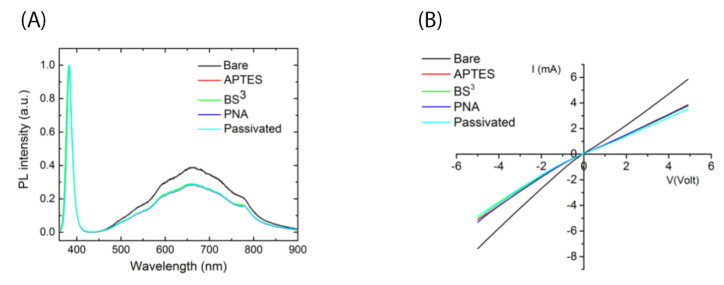
PL spectra (**A**) and I–V curves (**B**) of ZnONW-based device after each functionalization step.

In order to covalently immobilize the PNA probe on the ZnONW surface, the chemical procedure described in Section 2.7 was followed. Each functionalization step was monitored by both PL and electrical measurements. Figure 4A shows the PL spectra of ZnONWs under irradiation with UV light (325 nm). The PL spectrum of the ZnONWs is constituted by a near-band-edge peak at 380 nm, due to transitions of free carriers from the valence band to the conduction one (VB→CB), and a below-band-gap peak in the region of between 500 and 800 nm. The visible emission is ascribed to defects present both on the surface of the ZnONWs and inside the material, such as oxygen vacancies and Zn interstitials. Any variation of the near-band-edge peak at 380 nm was not observed, while quenching of the visible peak due to surface defects of about 27% was measured after the first functionalization step (i.e., silanization by APTES). The next steps did not induce other variations to the spectrum of the ZnONWs whose hydroxyl groups (OH) were already engaged in APTES molecules. An analog result was demonstrated by electrical characterization. Figure 4B reports the I–V curves of the ZnONW electrical device after each functionalization step; the slopes of the curves are the reciprocal of resistances of the device. The normalized resistance variations (ΔR_N_) were calculated as (R_i_–R_1_)/R_1_ with R_i_ resistance of the device after the *i-th* step of functionalization (i = 2, 3, 4, 5) and R1 resistance of the bare device after –OH activation. The ΔR_N_ values are summarized in Table 2.

The percentage of ΔR_N_ after APTES modification was 47%; negligible variations of the parameter ΔR_N_ were observed after the next functionalization steps.

**Figure 5:**
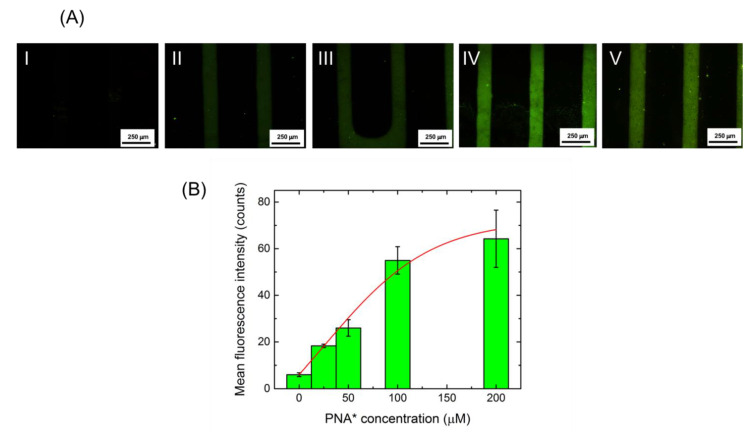
(**A**) Fluorescence microscopy imaging of ZnONW-based device before PNA immobilization (I) and after functionalization with 25 µM PNA* (II), 50 µM PNA* (III), 100 µM PNA* (IV), and 200 µM PNA* (V). (**B**) Mean fluorescence intensity of the device after functionalization with PNA* at different concentrations. Data were fitted using a dose-response curve (red line).

Since both the PL spectrum and I–V characteristic of the device did not show any variation after PNA immobilization, the saturation condition of the ZnONW surface with the PNA probe was investigated by fluorescence microscopy. To this aim, the ZnONW device was incubated with increasing concentrations (25, 50, 100, and 200 µM) of FAM-labeled PNA (PNA*). The corresponding fluorescence microscopy images are reported in Figure 5A, together with the mean fluorescence intensities (Figure 5B), calculated using the free Image J software (rel. 1.53a). Each fluorescence intensity was obtained averaging five independent values. Data were fitted using a dose-response curve (red-line) indicating that the saturation condition of the surface was reached for 100 µM PNA probe.

### 3.4. The Interaction between PNA-Modified ZnONWs and DNA 

**Figure 6:**
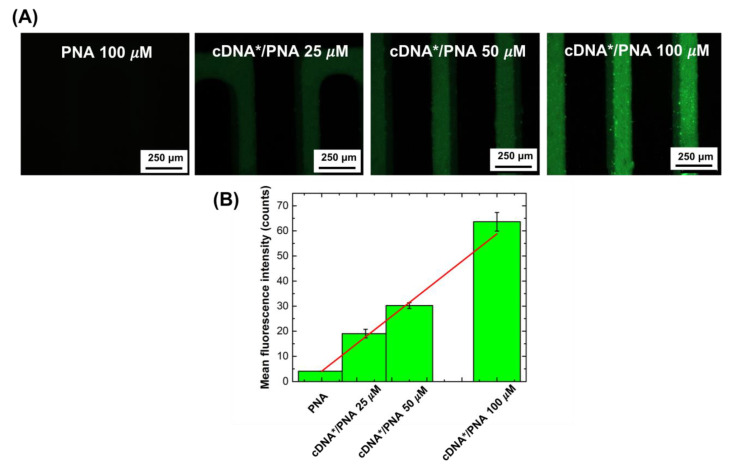
(**A**) Fluorescence microscopy imaging of ZnONW biochip surface after functionalization with 100 µM **PNA** probe and after incubation with 25, 50, and 100 µM cDNA*. (**B**) Mean fluorescence intensity, calculated from the images in (A), as a function of cDNA* concentration. Data were fitted using a linear regression model (red line).

Once PNA was covalently bound to the ZnONWs, its ability to interact with different concentrations of the specific complementary DNA (**cDNA**) was verified by electrical measurements, using a label-free PNA-modified ZnONW electrical transducer. Preliminary interaction experiments were performed by using the more robust optical analysis, which required the use of FAM-labeled cDNA (cDNA*). To this aim, both fluorescence microscopy images and photoluminescence spectra of ZnOWs functionalized with 100 µM of PNA were acquired before and after the incubation with increasing concentrations of cDNA* in the range included between 25 and 100 µM. The results of the fluorescence microscopy imaging reported in Figure 6 clearly demonstrated the effective hybridization. An increase in the fluorescence was observed with the increasing of cDNA* concentration. The values of the mean fluorescence intensity (Figure 6B), calculated from the images in Figure 6A using the software Image J, were fitted using a linear regression model (red-line in Figure 6A). The slope of the curve provides the sensitivity of such a nanostructured device to transduce the molecular interaction between **cDNA** and the PNA-ZnONWs, as 0.56 ± 0.02 counts µM^−1^. According to the IUPAC definition, the limit of detection (LOD) of this interaction can be defined as three times the ratio between the blank signal and the sensitivity. In this case, the blank was the sample functionalized with the PNA strand (i.e., the sample before cDNA* incubation). An LOD of 20 ± 1 µM was achieved considering a blank average intensity of 4.07 ± 0.05 counts, calculated from the first image of Figure 6A. The PL emission of modified ZnONWs was also studied before and after incubation with cDNA*. It was observed that the presence of cDNA* annealed with **PNA** did not induce a variation of the spectrum, except for the presence of a weak peak attributed to the fluorophore, circled in green in Figure S5. This result revealed that the PL emission of PNA-modified ZnONWs was not sensible to variations of surface charge due to the recognition of negatively charged DNA.

**Figure 7:**
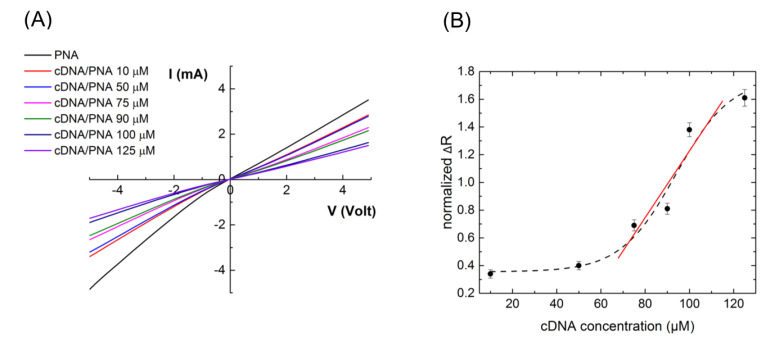
(**A**) I–V characteristics of ZnONW-based electrical transducer functionalized with 100 uM **PNA** probe, before and after interaction with increasing concentrations of **cDNA** in the 10 to 125 µM range. (**B**) Normalized ΔR vs. **cDNA** concentration. Data were fitted using a dose-response model (black dash curve); data included between 75 and 100 µM were fitted by a linear model (red curve).

After verifying the hybridization using cDNA*, some electrical measurements were also performed using label-free **cDNA**. The PNA-ZnONW electrical transducer, functionalized with 100 µM of **PNA** probe, was exposed to increasing concentrations of complementary DNA (**cDNA**), from 10 to 125 µM. The interaction between PNA-ZnONWs and **cDNA** was monitored by acquiring the I–V characteristics of the device. I–V curves, reported in Figure 7A, show that the **PNA/cDNA** hybridization induced an increase of the sensor resistance dependent on the DNA concentration increasing. Any variation was not observed by exposing the device to PBS 1× without DNA (data not shown here); the negative control confirmed than ZnONWs did not react with salts contained in the buffer solution. The normalized resistance variations, calculated by I–V curves, are reported in Figure 7B as a function of **cDNA** concentration; experimental data were fitted using a dose-response model (black dash curve). The electrical sensitivity of the nano-transducer in monitoring the **PNA/cDNA** interaction was calculated in the linear region of its response, between 75 and 100 µM, as the slope of the curve (red line); a value of 0.024 ± 0.014 µM^−1^ was determined. The ΔR_N_ of blank was the normalized resistance variation obtained by I–V curves of the biosensor, acquired before and after the exposure to water for 2 h at 4 °C; the ΔR_N_ of blank was calculated as 0.03 ± 0.01. In this case, the electrical LOD in monitoring the **PNA/cDNA** hybridization was achieved as 4 ± 2 µM. The result demonstrated that, due to the high electron mobility of ZnONWs, the PNA-modified ZnONWs show better performance as electrical transducers than as platforms for optical sensing, validating the label-free electrical read-out, the only one useful in the direct recognition of native DNA. Moreover, the sensitivity and LOD of the device are expected to be further improved, modifying the electrode geometry and decreasing the resistance of the nanowires by using a proper doping strategy [27,28].

### 3.5. PNA-ZnONWs Transducer Specificity

**Figure 8:**
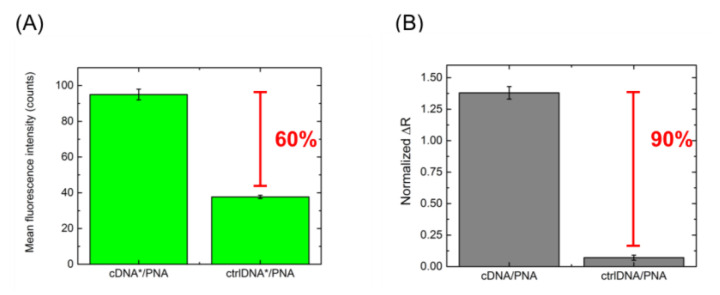
(**A**) Mean fluorescence intensity values of ZnONW-based devices after interaction with 100 µM cDNA* and 100 µM ctrlDNA*. (**B**) Normalized ΔR calculated from I–V curves of ZnONW-based devices after interaction with 100 µM **cDNA** and 100 µM **ctrlDNA**.

The PNA-modified ZnONW electrical transducer was exposed to 100 µM **cDNA** and 100 µM **ctrlDNA** to verify the specificity of the nanosystem. The biorecognition experiment was performed using both labeled and label-free sequences in order to compare the results of the fluorescence microscopy imaging with the results of the electrical characterization. Figure 8A reports the values of the mean fluorescence intensity calculated from the fluorescence images of the devices incubated with cDNA* and ctrlDNA* (data not shown here); the difference between the signals is 60%. The normalized resistance variations (ΔR_N_) calculated by the I–V curves of the sensor incubated with unlabeled **cDNA** and **ctrlDNA** (data not shown) were reported in Figure 8B. The results of the electrical characterization confirmed the optical data, but, in this case, the difference between the signals calculated using **cDNA** and **ctrlDNA** was about 90%, demonstrating that the PNA-modified ZnONW device is more specific when electrically interrogated. This could be ascribed to the higher stability of the heteroduplex **PNA/cDNA** obtained in the absence of the FAM fluorochrome, as demonstrated in Section 3.1.

## 4. Conclusions

Currently, the standard analytical technique used to diagnose LLC is represented by flow cytometry analyses whose instrumentation is expensive and requires highly specialized and trained personnel. The same considerations in terms of instrumentation cost and personnel requirements could also be applied to the PCR-based analytical techniques, even if both are highly sensitive and specific. Rapid, cost-effective, and easy-to-use tools for the early diagnosis of largely diffused diseases could improve the prognosis and the quality of life of patients. 

Here, we report a proof-of-concept study aimed at the development of an electrical transducer based on PNA-modified ZnONWs for the label-free monitoring of the DNA sequence (**cDNA**) corresponding to a short fragment of the mRNA of CD5, a prognostic marker of the chronic lymphatic leukemia. The nanostructured transducer is able to electrically detect the DNA sequences without sample-labeling. The interaction between the PNA probe immobilized on the surface of the electrical transducer and the DNA target induces a concentration-dependent variation in the resistance of the device that allows the detection of the molecular interaction down to 4 µM **cDNA**. It has also been demonstrated that the device can discriminate between complementary and non-complementary DNA sequences with a specificity of 90%. These encouraging results endorse the use of PNA-modified ZnONWs for the label-free electrical sensing of DNA/RNA fragments in disease diagnosis. However, further studies using longer PNA probes and RNA complementary and scrambled target sequences are needed to translate the proposed methodology from proof-of-concept to prototype devices. 

## Figures and Tables

**Table 1:** DNA and PNA sequences used in this study.

Sample	Sequence
**PNA**	AcNH-tttctctcccaa-KK-NH_2_ (N to C)
PNA*	FITC-(AEEA)_2_-tttctctcccaa-KK-NH_2_ (N to C)
**cDNA**	TTGGGAGAGAAA (5′-3′)
cDNA*	6FAM-TTGGGAGAGAAA (5′-3′)
**ctrlDNA**	CCTTTTTTTTTT (5′-3′)
ctrlDNA*	6FAM-CCTTTTTTTTTT (5′-3′)

**Table 2:** Calculated ΔR_N_ values after each functionalization step.

Functionalization Step	ΔR_N_
**APTES**	0.47 ± 0.03
**BS^3^**	0.52 ± 0.04
**PNA**	0.46 ± 0.04
**Passivated**	0.59 ± 0.04

## Data Availability

Data in this paper is available on the reasonable request upon the corresponding author.

## Supplementary Materials

The following are available online at https://www.mdpi.com/2079-4991/11/2/523/s1, Figure S1. ESI-MS spectra of PNA and DNA samples; Figure S2. Functionalization scheme of ZnONWs surface; Figure S3. CD melting profiles and Tm value of **cDNA/PNA** and **cDNA*/PNA**; Figure S4. PAGE mobility of FAM-labelled **cDNA** and **ctrlDNA** before and after incubation with **PNA**; Figure S5. PL spectra of PNA-ZnONWs before and after incubation with 100 µM **cDNA***.
