# Supplementary material for: Bioconjugation of a PNA Probe to Zinc Oxide Nanowires for Label-Free Sensing
Source: Nanomaterials (Basel). 2021 Feb 18;11(2):523. doi: 10.3390/nano11020523 (PMC7923052; doi:10.3390/nano11020523)
Supplement: Supplementary file 1 [file nanomaterials-11-00523-s001.zip › nanomaterials-1086001-supplementary.pdf]

SUPPORTING INFORMATION
of
Bioconjugation of a PNA Probe to Zinc Oxide Nanowires for Label-Free Sensing

Teresa Crisci[1,2], Andrea Patrizia Falanga[3], Maurizio Casalino[1], Nicola Borbone[4], Monica Terracciano[4], Giovanna Chianese[1], Mariano Gioffrè[1], Stefano D'Errico[4], Maria Marzano[5], Ilaria Rea[1*], Luca De Stefano[1§], and Giorgia Oliviero[3§]

[1] National Research Council, Institute of Applied Sciences and Intelligent Systems, Unit of Naples, 80131 Naples, Italy
[2] Department of Mathematics and Physics, University of Campania Luigi Vanvitelli, 81100 Caserta, Italy
[3] Department of Molecular Medicine and Medical Biotechnology, University of Naples Federico II, 80131 Naples, Italy
[4] Department of Pharmacy, University of Naples Federico II, 80131 Naples, Italy
[5] National Research Council, Institute of Crystallography, 70126, Bari, Italy

* Corresponding Author: ilaria.rea@na.isasi.cnr.it

§ These authors equally contributed to this work.

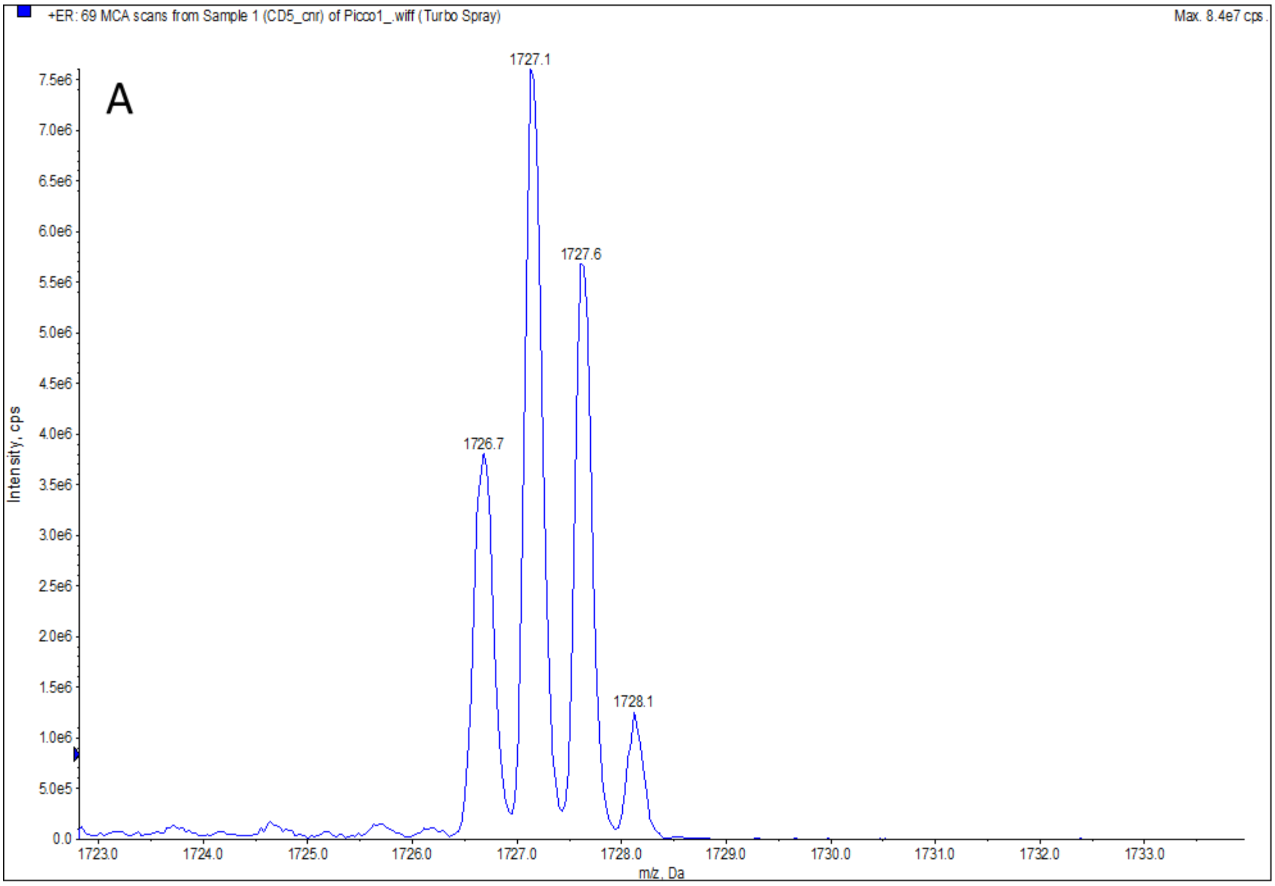

+ER: 69 MCA scans from Sample 1 (CD5_cnr) of Picco1_.wiff (Turbo Spray)
Max. 8.4e7 cps.
A
1727.1
1727.6
1726.7
1728.1
Intensity, cps
m/z, Da

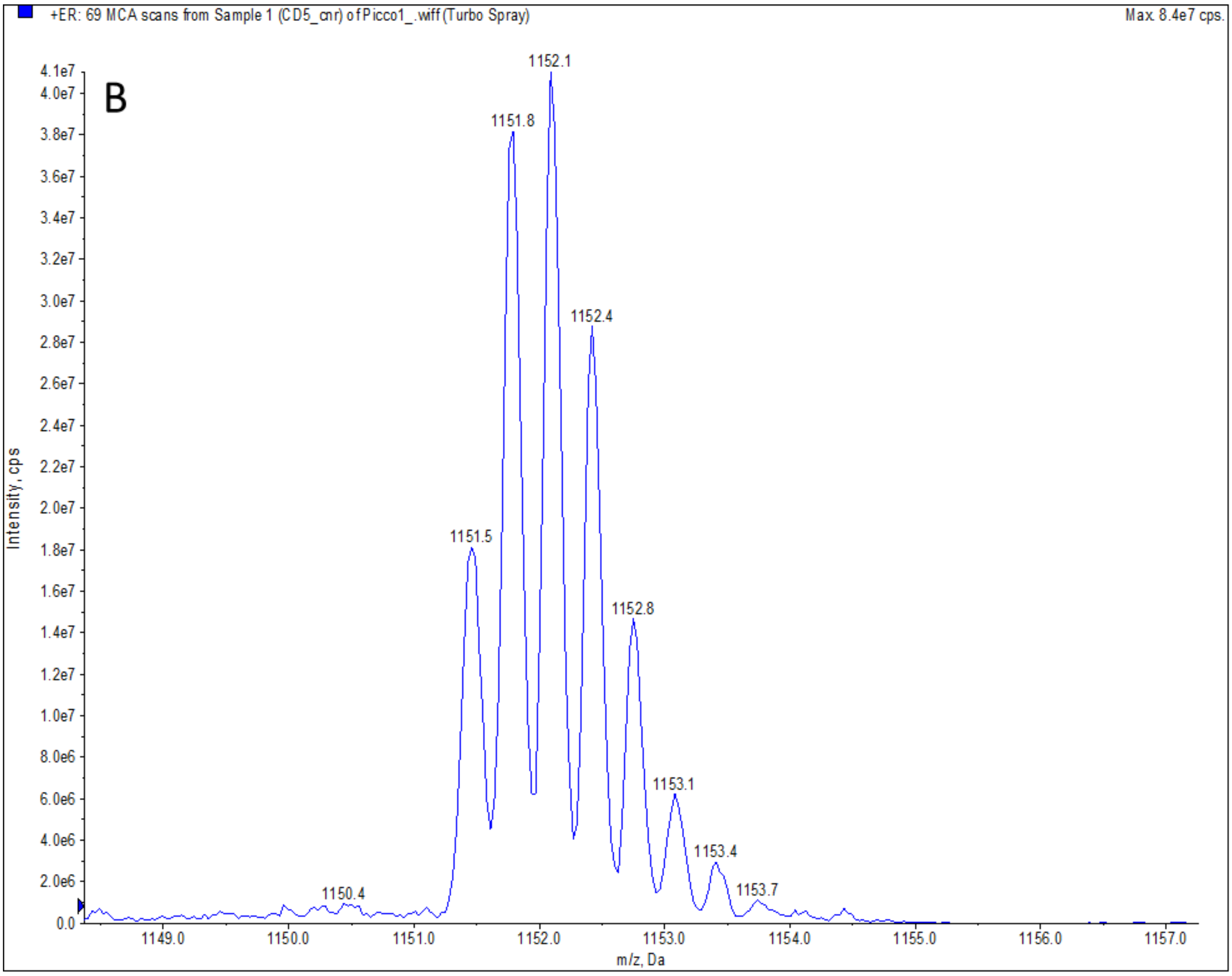
+ER: 69 MCA scans from Sample 1 (CD5_cnr) of Picco1_.wiff (Turbo Spray)
Max. 8.4e7 cps.
B
Intensity, cps
m/z, Da
1152.1
1151.8
1152.4
1151.5
1152.8
1153.1
1153.4
1153.7
1150.4

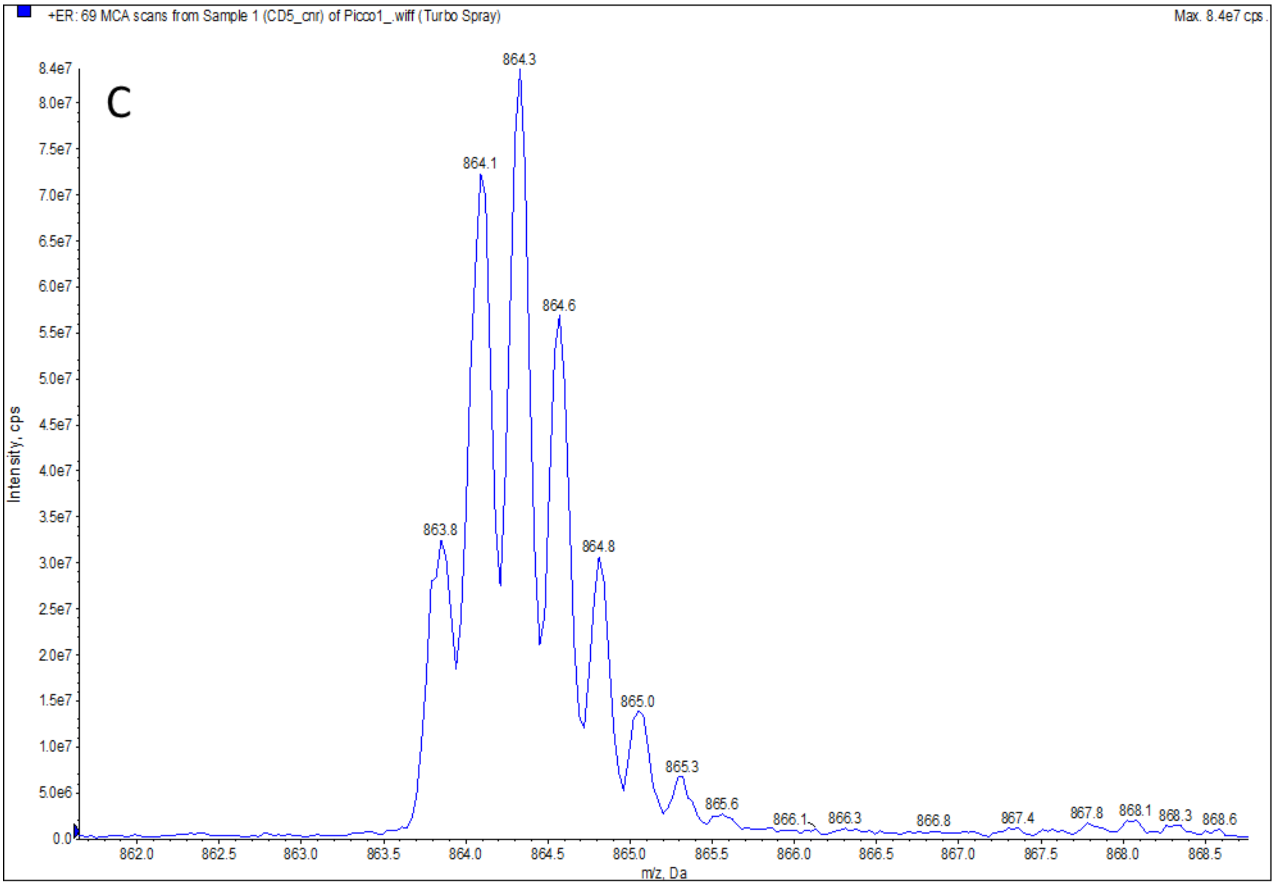
+ER: 69 MCA scans from Sample 1 (CD5_cnr) of Picco1_.wiff (Turbo Spray)
Max. 8.4e7 cps.
C
Intensity, cps
m/z, Da
863.8
864.1
864.3
864.6
864.8
865.0
865.3
865.6
866.1
866.3
866.8
867.4
867.8
868.1
868.3
868.6

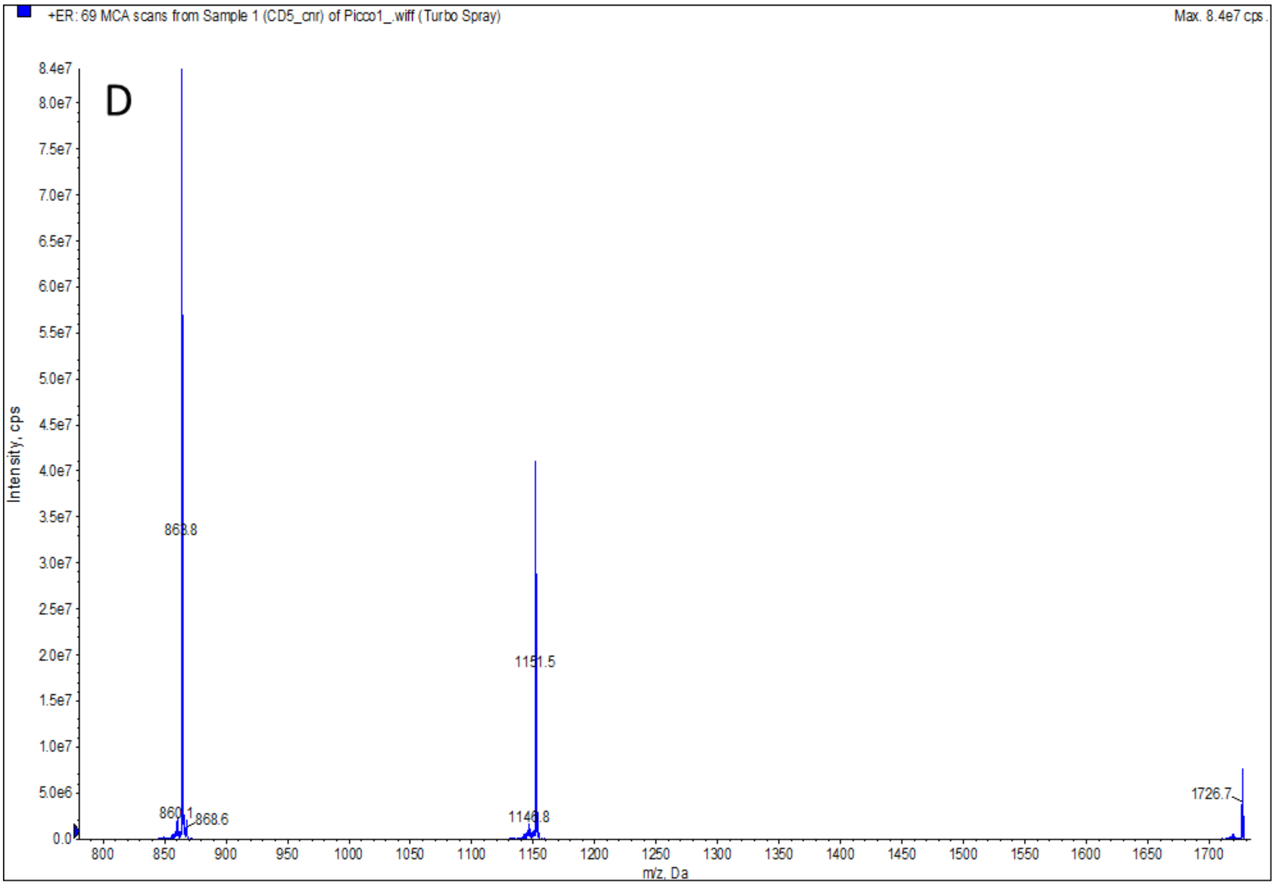
+ER: 69 MCA scans from Sample 1 (CD5_cnr) of Picco1_.wiff (Turbo Spray)
Max. 8.4e7 cps.
D
Intensity, cps
m/z, Da
868.6
1151.5
1726.7

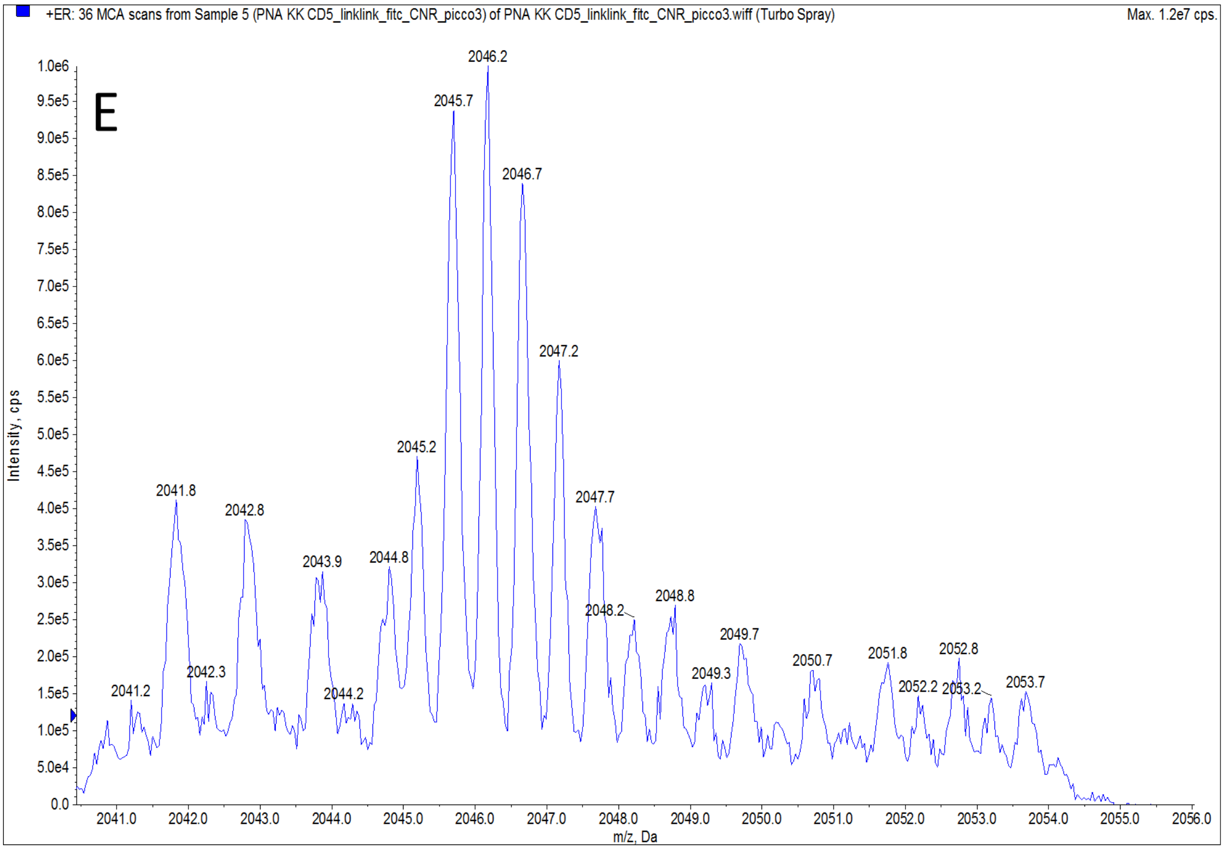
+ER: 36 MCA scans from Sample 5 (PNA KK CD5_linklink_fitc_CNR_picco3) of PNA KK CD5_linklink_fitc_CNR_picco3.wiff (Turbo Spray)
Max. 1.2e7 cps.
E
Intensity, cps
m/z, Da
2046.2
2045.7
2046.7
2047.2
2045.2
2041.8
2042.8
2047.7
2043.9
2044.8
2048.8
2048.2
2049.7
2042.3
2049.3
2050.7
2051.8
2052.8
2041.2
2044.2
2052.2
2053.2
2053.7

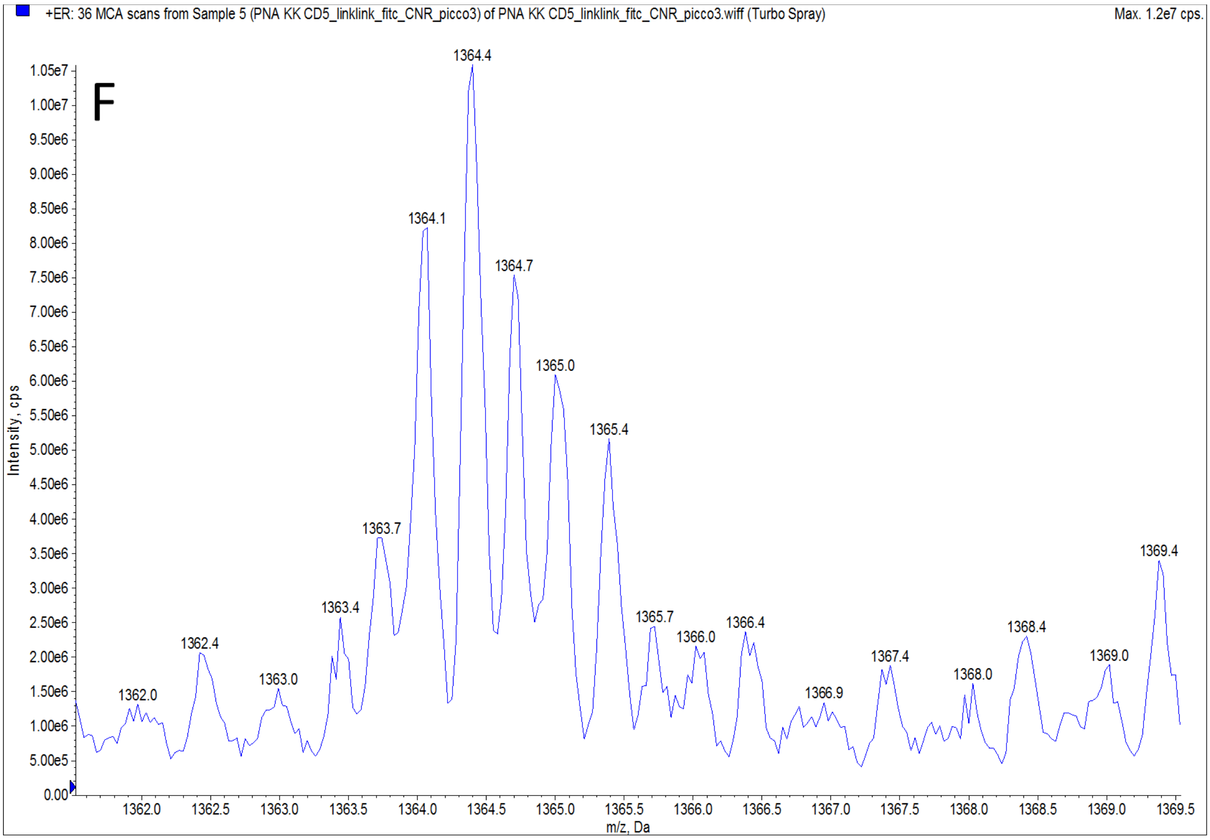
+ER: 36 MCA scans from Sample 5 (PNA KK CD5_linklink_fitc_CNR_picco3) of PNA KK CD5_linklink_fitc_CNR_picco3.wiff (Turbo Spray)
Max. 1.2e7 cps.
F
Intensity, cps
m/z, Da
1364.4
1364.1
1364.7
1365.0
1365.4
1363.7
1369.4
1363.4
1365.7
1366.4
1362.4
1366.0
1368.4
1367.4
1369.0
1368.0
1363.0
1362.0
1366.9

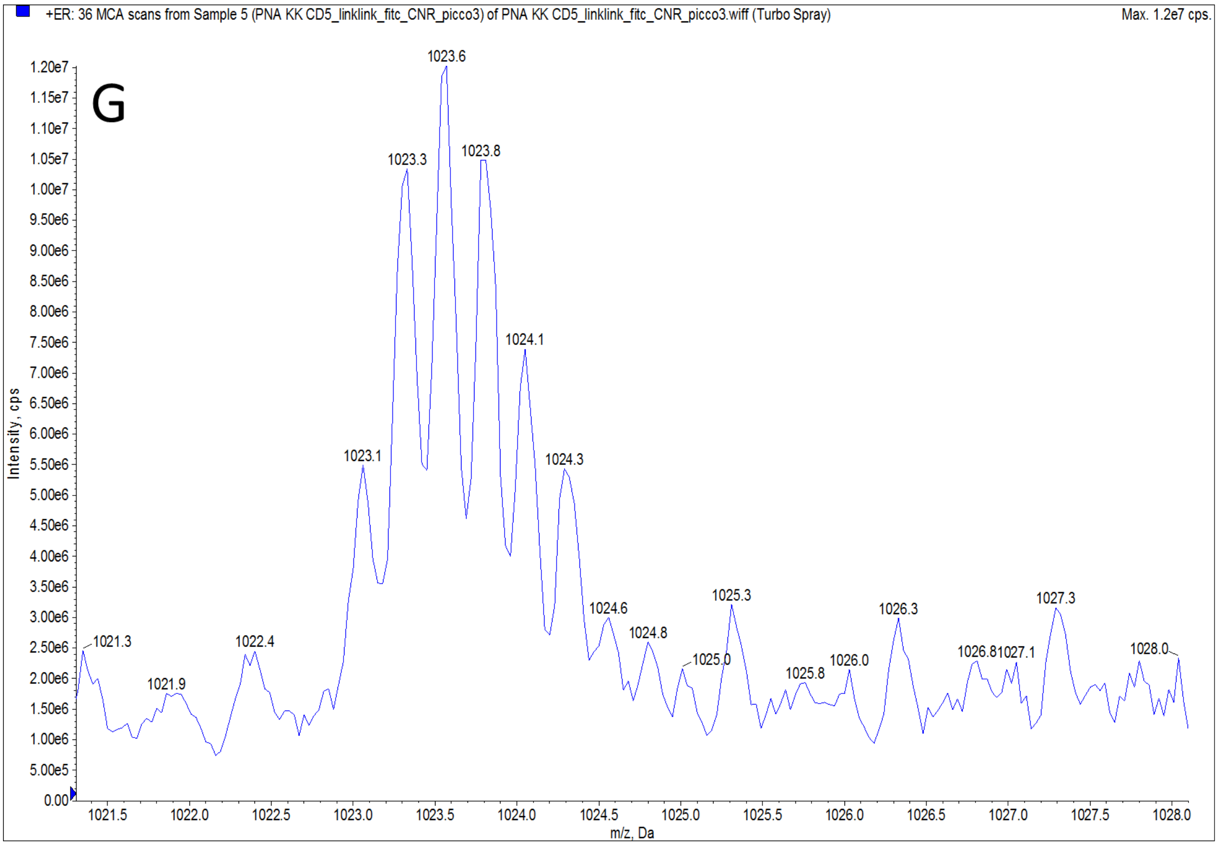
+ER: 36 MCA scans from Sample 5 (PNA KK CD5_linklink_fitc_CNR_picco3) of PNA KK CD5_linklink_fitc_CNR_picco3.wiff (Turbo Spray)
Max. 1.2e7 cps.
G
Intensity, cps
m/z, Da
1023.6
1023.3
1023.8
1024.1
1023.1
1024.3
1024.6
1024.8
1025.3
1025.0
1025.8
1026.0
1026.3
1026.8
1027.1
1027.3
1028.0
1021.3
1021.9
1022.4

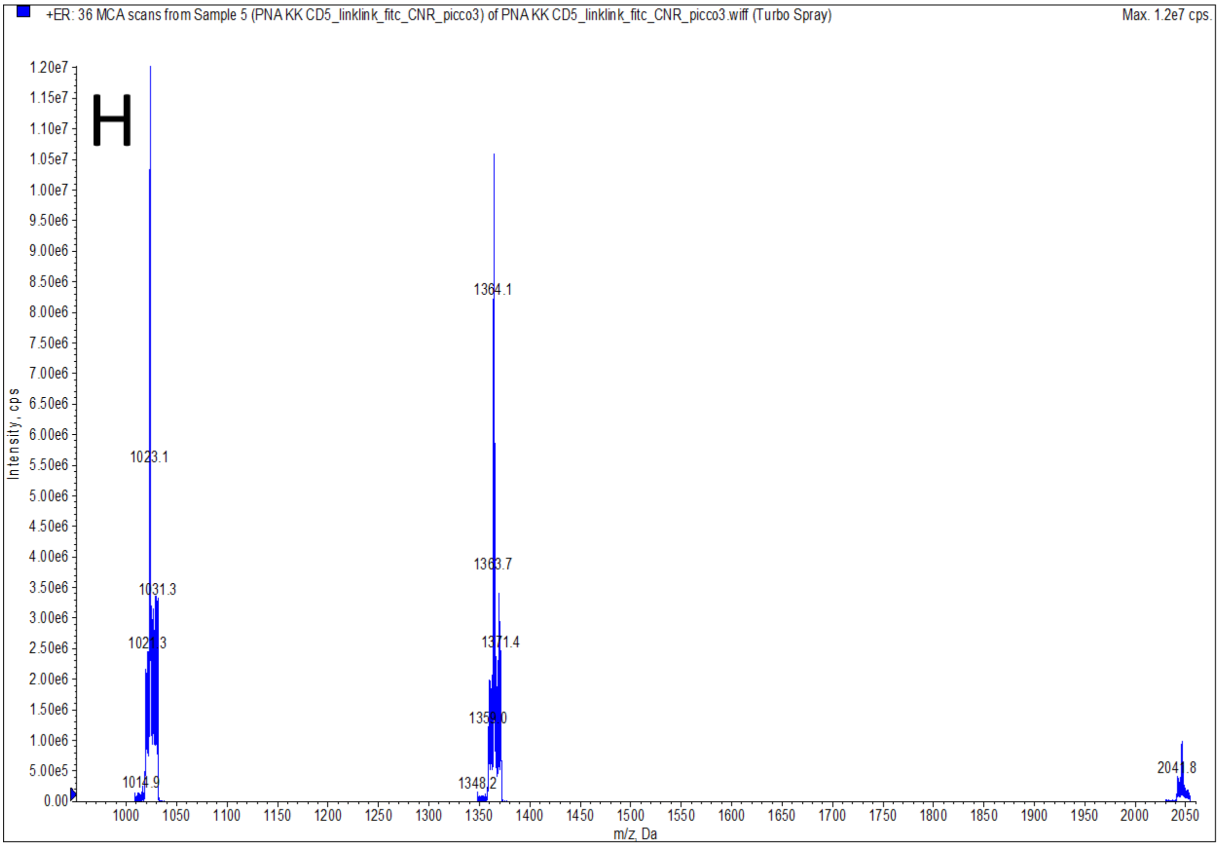
+ER: 36 MCA scans from Sample 5 (PNA KK CD5_linklink_fitc_CNR_picco3) of PNA KK CD5_linklink_fitc_CNR_picco3.wiff (Turbo Spray)
Max. 1.2e7 cps.
H
Intensity, cps
m/z, Da
1364.1
1363.7
1371.4
1348.2
2041.8

Figure S1: (A-D) **PNA:** Expansions of the MCA ESI-MS spectrum (m/z) calcd. for $[M + H]^+$ 3452.46, calcd. for $[M + 2H]^{2+}$ 1726.73, found 1726.7, calcd. for $[M + 3H]^{3+}$ 1151.49, found 1151.5; calcd. for $[M + 4H]^{4+}$ 863.86, found 863.8. (E-H) **PNA***: Expansions of the MCA ESI-MS spectrum (m/z) calcd. for $[M + H]^+$ 4089.64; calcd. for $[M + 2H]^{2+}$ 2045.28, found 2045.2; calcd. for $[M + 3H]^{3+}$ 1363.86, found 1363.7; calcd. for $[M + 4H]^{4+}$ 1023.14, found 1023.1.

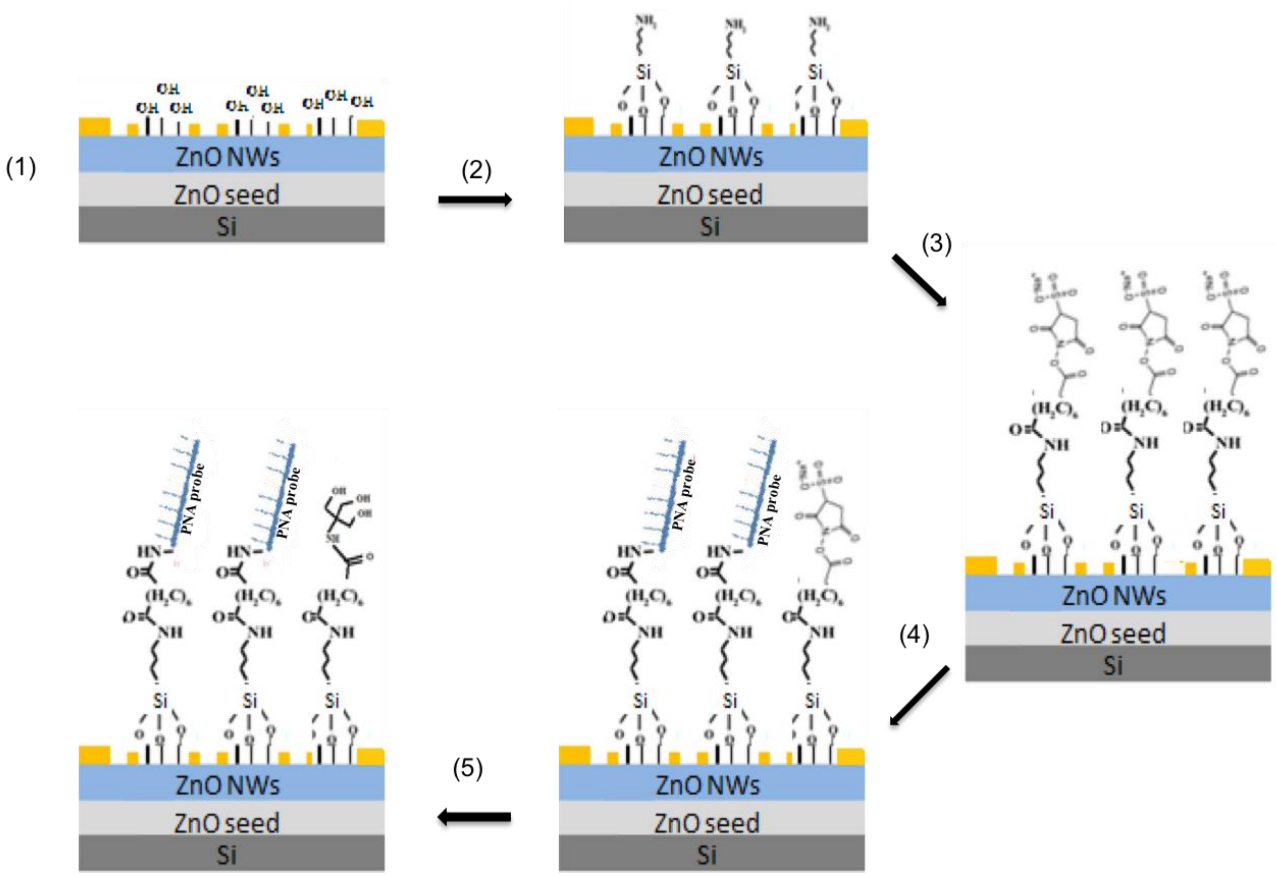

Figure S2. Functionalization scheme of ZnONWs surface. (1) Oxygen plasma. (2) Silanization by APTES. (3) BS^3 crosslinker modification. (4) PNA probe immobilization. (5) Surface passivation.

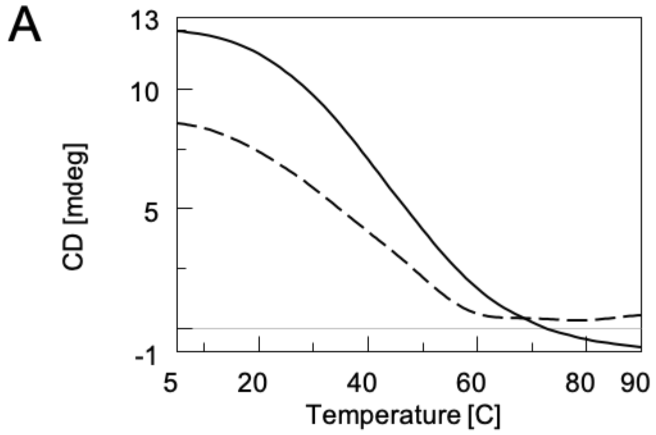

B

Sample	Tm (°C)
**cDNA/PNA**	45
**cDNA*/PNA**	40

Figure S3. (A) CD melting profiles of **cPNA/DNA** mixture (solid line) and **cDNA*/PNA** (dashed line) at 1:1 ratio. The curves were obtained by monitoring the absorbance at 266 nm, at a heating rate of 0.5 °C/min; (B) Table with Tm values of each sample.

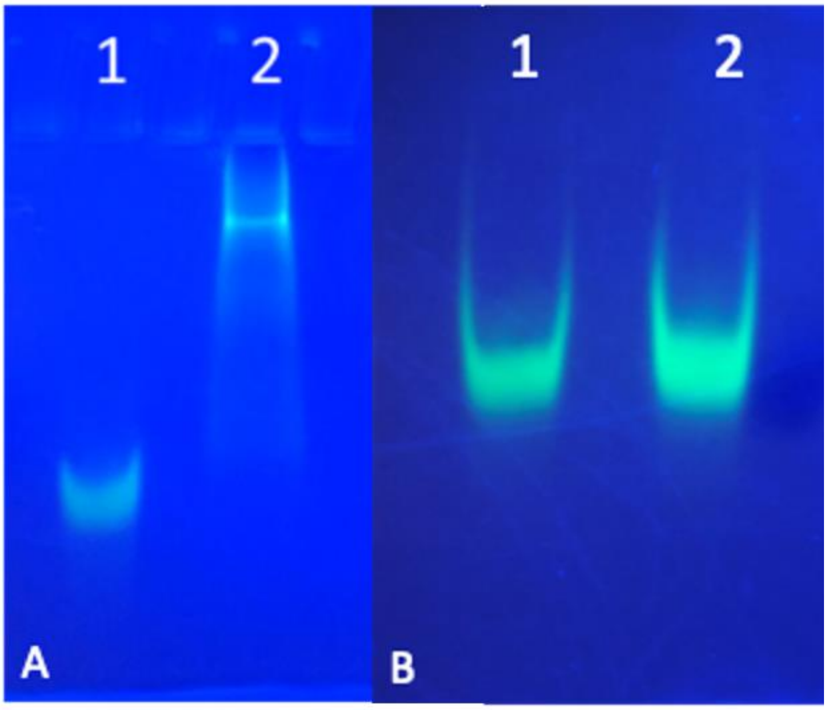

Figure S4. PAGE of **cDNA*** (panel A) or **ctrlDNA*** (panel B) annealed alone (lanes 1) or with **PNA** (1:1) (lanes 2) in 100 mM PBS solution.

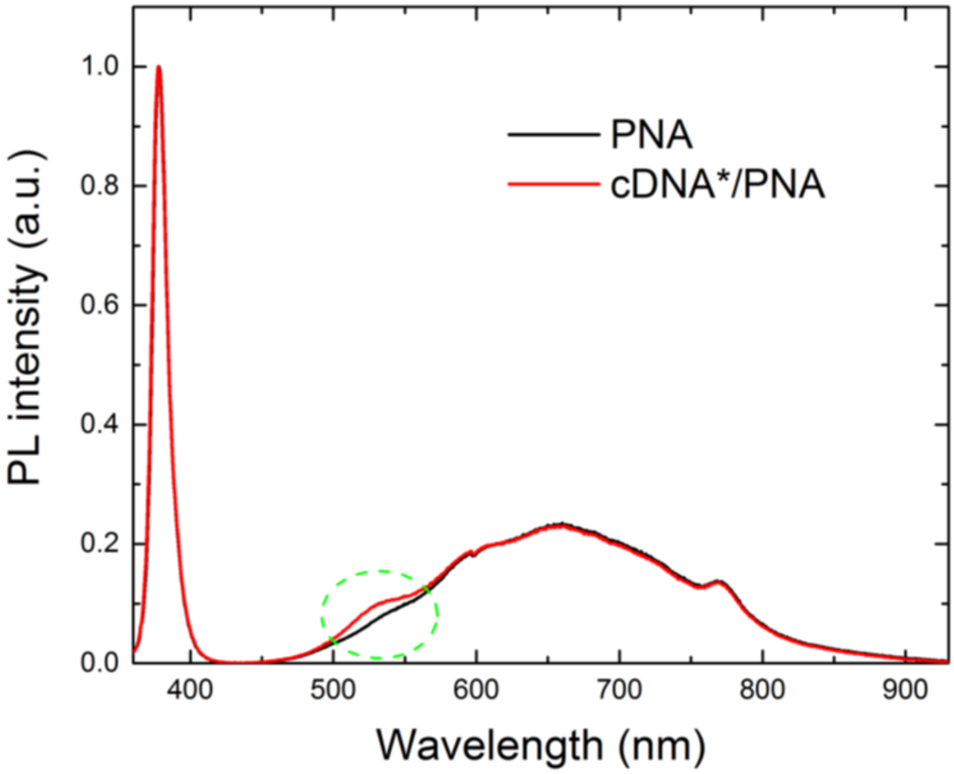

Figure S5. PL spectra of ZnONWs after PNA immobilization (black curve) and after interaction with 100 µM cDNA* (red line).
